# Trps1 Regulates Biliary Epithelial-Mesenchymal Transition and Has Roles during Biliary Fibrosis in Liver Grafts: A Preliminary Study

**DOI:** 10.1371/journal.pone.0123233

**Published:** 2015-04-17

**Authors:** Cheng Zhe, Fan Yu, Ju Tian, Shuguo Zheng

**Affiliations:** Institute of Hepatobiliary Surgery, Southwest Hospital, Third Military Medical University, No. 29 Gaotanyan Road, Shapingba District, Chongqing, 400038, China; Osaka University Graduate School of Medicine, JAPAN

## Abstract

**Objective:**

To investigate the role(s) of Trps1 in non-anastomotic biliary stricture (NABS) following liver transplantation.

**Methods:**

Immunohistochemical and histological techniques were used to detect Trps1, E-cadherin, CK19, vimentin, α-SMA, and collagen deposition. Human intrahepatic biliary epithelial cells (HIBECs) were infected with a Trps1 adenovirus, or transfected with Trps1 short-interfering RNAs (siRNAs). Reverse transcription polymerase chain reaction (RT-PCR) assays and western blotting were used to determine expression levels of epithelial and mesenchymal markers, and Trps1 in HIBECs.

**Results:**

Expression of Trps1 and epithelial markers was down-regulated or absent in NABS liver samples. Mesenchymal markers were seen in biliary epithelial cells (BECs), with collagen deposited around the bile duct. Trps1 expression positively correlated with epithelial markers. Expression of epithelial marker mRNAs and proteins in HIBECs decreased with prolonged cold preservation (CP), while mesenchymal marker expression increased. A 12-h CP period led to increased Trps1 mRNA and protein levels. Expression of E-cadherin was increased in HIBECs following Trps1 adenovirus infection and CP/reperfusion injury (CPRI), with vimentin expression levels reduced and CPRI-mediated epithelial-mesenchymal transition (EMT) inhibited. Transfection of HIBECs with Trps1 siRNAs in conjunction with CPRI revealed that E-cadherin expression was decreased, vimentin expression was increased, and CPRI-mediated EMT was promoted.

**Conclusion:**

Trps1 is involved in NABS pathogenesis following liver transplantation and negatively correlates with BEC EMT and biliary fibrosis in liver grafts. Trps1 demonstrates antagonistic effects that could reverse EMT.

## Introduction

Non-anastomotic biliary stricture (NABS) is a major biliary complication after liver transplantation, and can result in biliary cirrhosis, graft failure, re-transplantation, or death. NABS is a significant bottleneck that limits the improvement of liver transplantation efficacy. Pathologically, NABS progresses with biliary fibrosis of the graft, resulting in ineffective or excessive repair of the injured bile duct epithelium. Cold preservation/reperfusion injury (CPRI) is an independent factor of biliary fibrosis following liver transplantation, however the mechanisms involved require clarification [1].

Previous studies into the mechanisms of biliary fibrosis in liver grafts have primarily focused upon the pathway of TGF-β-mediated hepatic stellate cell activation. Cells that synthesize matrix are derived from the inherent source (activated hepatic stellate cell) and other heterologous cell populations [2]. Hepatocyte or biliary epithelial cells (BECs) are the major matrix-synthesizing cells in liver and biliary tract fibrosis during epithelial-mesenchymal transition (EMT) [3].

EMT is closely related to embryogenesis, injury repair, organ fibrosis, and tumor invasion/metastasis. EMT has been shown to promote organ fibrosis in multiple studies [4, 5]. During the development of primary biliary cirrhosis and primary sclerosing cholangitis, EMT of bile duct epithelial cells is a major source of matrix-producing cells, and is a critical factor in liver and biliary tract fibrosis [6, 7]. *In vitro* studies have confirmed that mature BECs can undergo EMT in the presence of particular pro-inflammatory cytokines [8]. Liu X *et al*. [9] observed EMT of BECs during biliary fibrosis following orthotopic liver transplantation in Sprague-Dawley rats.

The development and progression of EMT requires specific micro-environmental conditions and precise regulation of intracellular signals. Certain signaling molecules, including transforming growth factor (TGF)-β, Notch, Wnt, matrix metalloproteinases, Snail, Twist, and Zeb have been shown to stimulate EMT [10, 11]. Several other signaling molecules, such as tricho-rhino-phalangeal syndrome type 1 (Trps1) and bone morphogenetic protein 7 (BMP-7) have been shown to inhibit EMT [12, 13]. Trps1 is an atypical GATA transcription factor that specifically binds to the GATA motif of the Trps1 gene. It competitively inhibits Trps1 expression that is activated by other GATA transcription factors (GATA-1, -2, and -3) [14]. Recent studies have shown that Trps1 is involved in antagonizing EMT in renal tubular epithelial cells [15], alveolar epithelial cells [16], and breast cancer cells [17]. Whether Trps1 is involved in the negative regulation of BEC EMT, and inhibits biliary fibrosis in grafts following liver transplantation remains unknown. We sought investigate the role(s) of Trps1 in NABS following liver transplantation.

## Methods

### Patients and tissue samples

We used six paraffin-embedded secondary liver transplant samples, taken from January 2010 to June 2012, that were histopathologically diagnosed with biliary fibrosis of the graft at the Southwest Hospital of the Third Military Medical University. A summary of patient characteristics are shown in Table 1, and all diagnoses were made by a full-time pathology doctor. Normal bile duct surrounding hepatic cavernous hemangioma tumor tissue was used as a control (7 cases). We obtained patient consent and approval from the Research Ethics Committee of the Southwest Hospital of the Third Military Medical University to use these clinical materials in our study.

**Table 1:** General summary of patient information.

ID	Sex	Age (years)	Time of primary liver transplantation	Time of secondary liver transplantation	Surgery method	Surgery duration (min)	Duration of cold ischemia (min)	Blood loss volume during the surgery (ml)
1	female	45	2009.10.30	2010.04.16	piggyback	321	420	1800
2	female	40	2006.09.22	2010.05.28	piggyback	560	630	4800
3	Male	45	2008.12.18	2010.08.02	piggyback	406	450	1000
4	male	47	2004.04.02	2011.01.14	piggyback	360	360	4000
5	male	41	2011.01.30	2011.03.11	conventional	496	550	6000
6	male	28	2010.03.15	2011.01.21	piggyback	413	500	3000

### Cell lines and reagents

Human intrahepatic biliary epithelial cells (HIBECs) were purchased from ScienCell Research Laboratories (CA, USA). Short-interfering RNAs (siRNAs) specific for Trps1 (sc106642) and control siRNAs (sc37007) were ordered from Santa Cruz Biotechnology (CA, USA). We obtained RPMI 1640, fetal bovine serum (FBS), and trypsin from HyClone (USA). The UW solution was from Bristol-Myers Squibb, Trizol was from Invitrogen, a ReverTra Ace® reverse transcription kit was from TOYOBO, RIPA lysis buffer was from Chongqing Jinmai Technology Co. Ltd (China), and agarose was bought from Takara. We purchased diethylpyrocarbonate (DEPC), phenylmethanesulfonyl fluoride (PMSF), and sodium dodecyl sulfate (SDS) from Sigma–Aldrich (USA).

### Masson’s trichrome staining

Specimens were fixed in 10% buffered formalin, embedded in paraffin, sectioned (4-μm thickness), and stained with hematoxylin and eosin. Paraffin sections were also stained with 1% (v/v) HCl and Masson’s composite staining solution (Sigma) was added drop-wise for 5–10 min. Sections were subsequently washed with distilled water, treated with 1% phosphotungstic acid, and incubated with aniline blue staining solution for 5–10 min. After treatment with 1% glacial acetic acid for 1 min and dehydration with 95% (v/v) alcohol, sections were mounted with neutral gum. Collagen fibers around the bile duct stained blue. For each sample, we randomly selected five fields of view at a high power (×200 magnification), and calculated the proportion of blue staining around the bile duct using Image-pro software.

### Immunohistochemistry

Sections were incubated with primary antibodies against *TRPS1* (diluted 1:200; sc-26975), E-Cadherin (1:400; sc-1500), CK19 (1:200; sc-33119), Vimentin (1:200; sc-7557), α-SMA (1:200, sc-324317), and β-catenin (1:500; sc-81178; all from Santa Cruz Biotechnology, CA, USA). The secondary antibodies used were from an immunohistochemistry staining kit (Zhongshan Biotechnology Company, Beijing, China). After diaminobenzidine (DAB) staining, sections were counterstained with hematoxylin. For negative controls, primary antibodies were replaced with phosphate-buffered saline (PBS). The presence of brown granules was considered a positive signal. Five fields of view at high magnification were selected for each section and 100 BECs assessed in each field of view.

### Cell culture and in vitro CPRI

HIBECs (P5100) were incubated at 37°C/5% CO_2_ and passaged while in log phase using 0.25% (w/v) trypsin. Replication of CPRI was achieved by placing cells at 4°C in UW solution for 12, 24, and 48 h, followed by transfer to regular culture medium containing serum, and then incubating at 37°C for 1 h [18].

### Cell infection and transfection

HIBECs were seeded in 6-well plates (2 × 10^5^ cells/well) and cultured for 24 h or until 60–80% confluent. Cells were infected with either recombinant adenovirus containing Trps1 using a multiplicity of infection (MOI) of 80, or an empty virus control. Virus was allowed to adsorb to cells for 2 h at 37°C/5% CO_2_ before the medium was replaced with complete RPMI 1640 containing FBS. Cells were maintained at 37°C/5% CO_2_ for another 48 h before cells were harvested. For siRNA transfection, HIBECs were plated in 6-well plates (2 × 10^5^ cells/well) and cultured for 24 h or until 60–80% confluent. We added Trps1 or control siRNAs at an MOI of 100 in the presence of 250 μl of serum-free, antibiotic-free RPMI 1640. Cultures were mixed gently and incubated at 37°C/5% CO_2_ for 6 h. Medium was replaced with RPMI 1640 containing serum and incubated for another 24 h before cells were harvested.

### Trps1 immunohistochemistry

Dry and sterile coverslips were placed in 6-well plates and HIBECs seeded (1 × 10^5^ cells/well) in 3 ml of medium containing 10% (v/v) FBS. Cultures were incubated at 37°C/5% CO_2_ until cells had attached to coverslips. The culture medium was aspirated and cells rinsed with D-Hank’s solution before fixing with acetone (4°C for 10 min). Coverslips were removed, dried, subjected to Trps1 immunohistochemistry and subsequent staining with DAB substrate according to a two-step protocol. Coverslips were thoroughly rinsed with tap water, re-stained, and dehydrated. Coverslips were sealed with mounting medium.

### Quantitative PCR (qPCR) assays

Total RNA was prepared from HIBECs using Trizol reagent. Reverse transcription of total RNA into cDNA was conducted using a ReverTra Ace RT-PCR kit according to the manufacturer’s instructions. Specific primers targeting Trps1, E-cadherin, CK19, Vimentin, and α-SMA were designed, using Gene Tool, against gene sequences in GenBank (Table 2). We used β-actin as an internal control reference gene. Primers were synthesized by Shanghai Jierui Bioengineering Co., Ltd. Amplification reactions were performed in a 20-μl volume containing 2 μl of cDNA template, 10 μl of 2× PCR Mix Taq, 10 pmol of forward primer, and 10 pmol of reverse primer. Amplicons were separated by electrophoresis at 120 V for 20 min on a 2% (W/V) agarose gel containing ethidium bromide and visualized under UV light. Images were acquired using Quantity One 4.5 analysis software (Bio-Rad) and band intensities determined. The ratio of the integral optical density (IOD) for each PCR product to the IOD of the β-actin amplicon was used to express the relative gene content in HIBECs.

**Table 2:** Oligonucleotide primer sequences used in this study and expected amplicon sizes.

Gene	Primer sequences	PCR product sizes (bp)
Trps1	forward primer: 5′-TTGAGAGAGACACTACATGAC-3′	256
	reverse primer: 5′-TGTTAAAGATACTTTTTTCCC-3′	
E-cadherin	forward primer:5′- GGACCACAGGCATGCACCACTAC -3′	162
	reverse primer:5′- GGCAGGTGCAGTGGCTCATGT -3′	
CK-19	forward primer: 5′- CCCGCGACTACAGCCACTACTAC -3′	176
	reverse primer: 5′- GTCGGCCTCCACGCTCATG -3′	
Vimentin	forward primer: 5′-CCAGGCAAAGCAGGAGTCCAC-3′	247
	reverse primer: 5′-GGCCATCTTAACATTGAGCAGGT-3′	
α-SMA	forward primer: 5′- GGGCTAAGTTCTGTGGGGTGTGC -3′	138
	reverse primer: 5′- GGCAGGCACAGGTCTTGATGAA -3′	
β-actin	forward primer: 5′-CCATGTACGTTGCTATCCTGGC-3′	289
	reverse primer: 5′-ATCTCTTGCTCGAAGTCCAGGG-3′	

### Western blotting

Total protein was prepared from HIBECs using cell lysis buffer, and quantified using Coomassie blue staining. An equal amount of protein per sample was loaded onto polyacrylamide gels, separated by SDS-polyacrylamide gel electrophoresis (PAGE), transferred to PVDF membranes, and blocked for 2 h with 5% (w/v) skim milk. Membranes were incubated with antibodies against Trps1, E-cadherin, CK19, Vimentin or α-SMA (1:200 dilution) at 4°C overnight. Membranes were then incubated with a horseradish peroxidase (HrP)-conjugated secondary antibody at 37°C for 2 h. Membranes were washed, incubated with chemiluminescent substrate for 5 min, and exposed to X-ray film. Bands were quantitatively analyzed using Quantity One 4.5 software. All experiments were repeated at least three times. Results are expressed as the intensity ratios for the proteins of interest against the internal control.

### Scratch assays

HIBECs were seeded into 6-well plates and mitomycin C (1 μg/ml) added to confluent cultured cells for 2 h to inhibit proliferation. Monolayers were then uniformly ‘scratched’ with the tip of a 1-ml pipet. Culture medium was replaced and cells left room temperature; after 12 h, the migratory distances of cells along the scratches were observed and velocities compared.

### Statistical analysis

We used SPSS 13.0 (SPSS Inc.) to perform two-sample comparisons using independent *t*-tests. Multiple-sample comparisons were performed using *χ* ^2^ tests. The correlation between two variables was analyzed using linear regression.

## Results

### HIBECs morphology and migration

**Fig 1:**
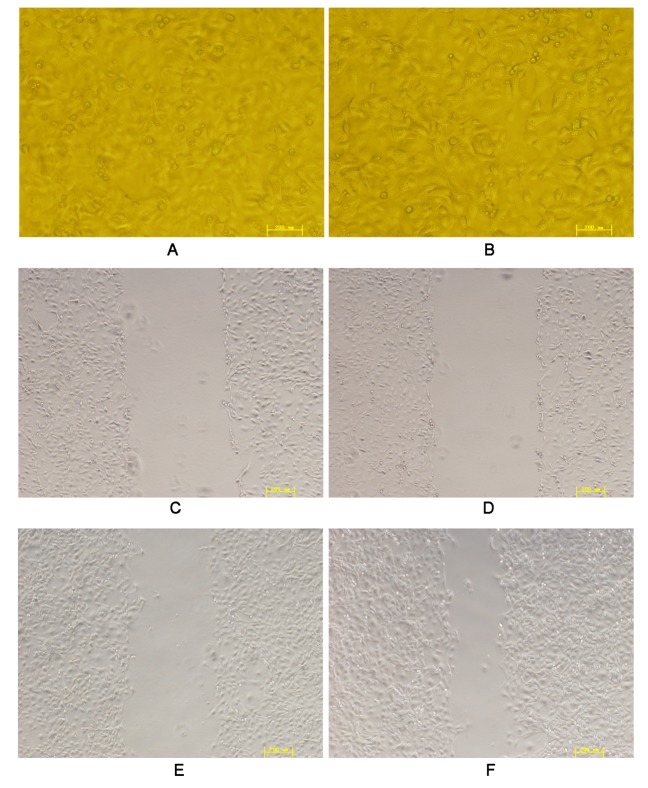
Scratch assays examining the effects of CPRI on the migratory capacity of HIBECs. A: normal HIBECs (×200 magnification); B: HIBECs subjected 24 h of CP and RI for 1 h (×200 magnification); C: 0 h after scratching normal HIBECs (×100 magnification); D: 0 h after scratching HIBECs subjected to CPRI (×100 magnification); D: 12 h after scratching normal HIBECs (×100 magnification); E: 12 h after scratching HIBECs subjected to CPRI (×100 magnification).

Following a 12-h CP period and 1 h of RI, normally ovoid HIBECs were transformed into fibroblast-like cells with a spindle morphology. Additionally, their cell migratory speed was significantly increased from 8.3 ± 2.2 μm/h to 32.5 ± 4.7 μm/h (*p* = 0.02; Fig 1).

### Immunohistochemistry and collagen deposition

**Fig 2:**
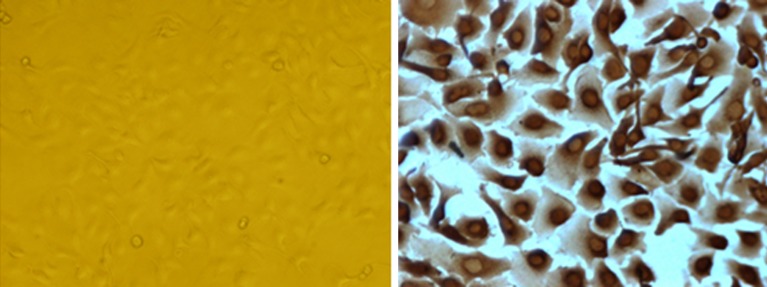
Trps1 expression in cultured HIBECs. Left: cultured HIBECs; Right: immunohistochemical staining of Trps1 in cultured HIBECs.

Positive staining for E-cadherin and CK19 was seen on the cellular membrane and in the cytoplasm. In control groups, epithelial markers were normally expressed in all BECs In the NABS group, expression of some of the epithelial markers was absent Vimentin and α-SMA were expressed at variable levels in the NABS group and not detected in the control group Trps1 was mainly expressed in the nuclei of BECs, with a small fraction expressed in the cytoplasm. In the control group, Trps1s was normally expressed in almost all BECs however in the NABS group, biliary epithelial Trps1 expression was less extensive (Fig 2). In the NABS group, bile duct hyperplasia was distinct, and a large amount of collagen was deposited around the bile ducts. There appeared to be severe damage to BECs as distinguished by altered cell morphologies. The BECs in the control group had normal morphologies and very little collagen deposited around the bile duct. Samples from NABS patients exhibited significantly reduced expression levels of E-cadherin, CK-19, Vimentin, α-SMA, and Trps1 (*P* < 0.01; Table 3) in comparison with those for the control group. Significant differences in biliary fibrosis were also seen between the two groups (*P* < 0.01), with NABS samples having more severe bile duct fibrosis (Table 4). In NABS samples, Trps1 expression levels positively correlated with epithelial marker expression, and negatively correlated with mesenchymal marker expression, and degree of biliary fibrosis in the liver graft (Table 5).

**Table 3:** Immunohistochemical staining rates in NABS samples for epithelial and mesenchymal markers, and Trps1.

ID	age(years)	gender	E-cadherin	CK19	Vimentin	α-SMA	Trps1%	Collagen %
			%	%	%	%		
1	45	female	92	91	13	15	74	20
2	40	female	67	64	80	77	22	41
3	45	male	91	92	18	15	71	25
4	47	male	94	93	10	12	80	17
5	41	male	75	80	60	62	45	36
6	28	male	85	90	30	19	60	33

**Table 4:** Rates of biliary Trps1, epithelial markers, mesenchymal markers, and collagen deposition in NABS and control samples.

	Patient group (n = 6)	Control group (n = 7)	P value
E-cadherin(%)	84.00±10.81	95.63±4.08	0.000
CK19(%)	85.00±11.31	96.71±3.72	0.016
Vimentin(%)	35.17±28.54	6.78±5.33	0.001
α-SMA(%)	33.33±28.50	5.46±3.85	0.001
Trps1(%)	58.67±21.80	96.27±5.13	0.000
Collagen(%)	28.67±9.48	3.84±2.27	0.005

**Table 5:** Correlation analysis between Trps1 expression, and expression of epithelial markers and mesenchymal markers during biliary fibrosis.

	correlation coefficients r	P value	regression formulas
Trps1-E-cadherin	0.993	0.000	T = –109.621+2.003E
Trps1-CK19	0.968	0.001	T = –99.911+1.866C
Trps1-Vimentin	0.990	0.000	T = 85.249–0.756V
Trps1-α-SMA	0.958	0.003	T = 80.078–0.732S
Collagen-Trps1	0.942	0.005	C = 52.715–0.410T

### Trps1 expression in HIBECs

**Fig 3:**
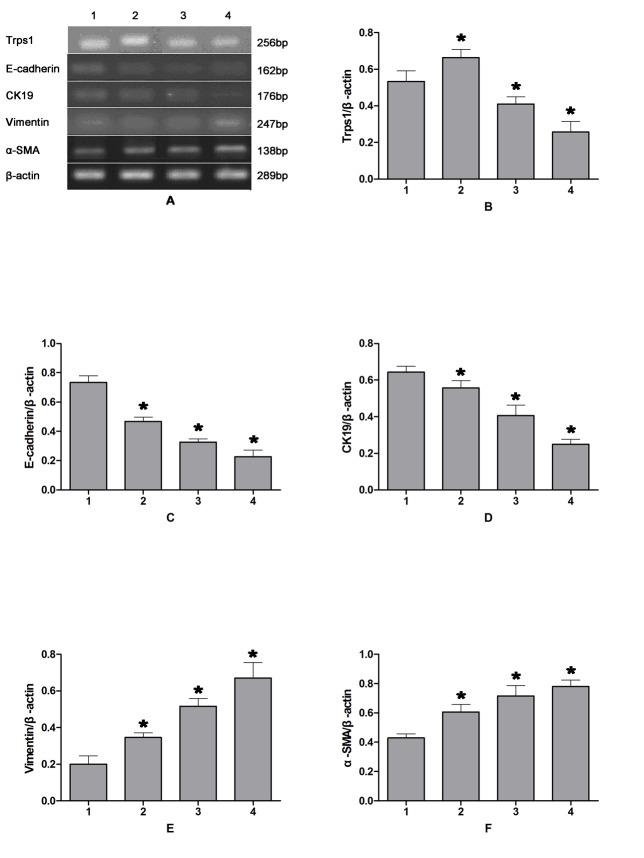
Changes in expression levels of Trps1 and EMT markers in HIBECs following CPRI. A: RT-PCR results; B–F: semi-quantitative PCR analysis of Trps1, E-cadherin, CK19, Vimentin, and α-SMA mRNA expression. Lane 1: control group; 2: CP for 12 h, RI for 1 h; 3: CP for 24 h, RI for 1 h; 4: CP for 48 h, RI for 1 h. CP, cold preservation. RI, reperfusion injury.

**Fig 4:**
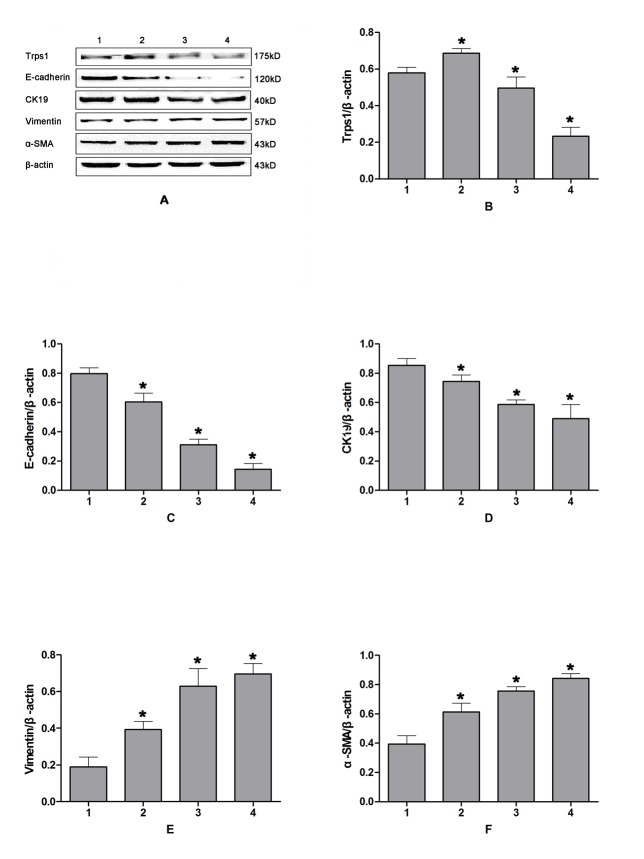
Western blotting analysis Trps1 expression changes and relevant EMT markers in HIBECs following CPRI. A: western blotting results; B–F: semi-quantitative measurement of Trps1, E-cadherin, CK19, Vimentin, and α-SMA protein expression levels. Lane 1: control group; 2: CP for 12 h, RI for 1 h; 3: CP for 24 h, RI for 1 h; 4: CP for 48 h, RI for 1 h. CP, cold preservation. R, reperfusion injury.

The Trps1 protein was primarily seen in the nucleus, and to a lesser extent in the cytoplasm, of cultured HIBECs (Fig 3). Following 12 h of CP, expression levels of the Trps1 gene and protein were significantly increased (*p* = 0.018 and 0.031, respectively) in HIBECs compared with those in untreated controls. In contrast, these were significantly decreased in HIBECs subjected to 24- or 48-h CP (p = 0.026 and 0.009, respectively), with a continued decrease observed after 48 h of CP (Fig 4).

### Epithelial/mesenchymal marker expression in HIBECs

In HIBECs subjected to 12, 24, or 48 h of CP, mRNA transcription and protein expression levels of E-cadherin were significantly decreased (*p* = 0.018 and 0.011, respectively), as were CK19 levels (*p* = 0.039 and 0.044, respectively). These decreases were more pronounced as the CP period was prolonged. In contrast, mRNA and protein expression levels in HIBECs subjected to CPRI were significantly increased for Vimentin (*p* = 0.002 and 0.026, respectively) and α-SMA (*p* = 0.035 and 0.016, respectively) compared with those in untreated controls. These differences became more distinct as the CP period was prolonged (Fig 4). Trps1 mRNA expression levels positively correlated with those for epithelial markers (E-cadherin and CK19), and negatively correlated with those for mesenchymal markers (Vimentin and α-SMA; Table 6).

**Table 6:** Correlation of Trps1 mRNA and EMT mRNA transcript expression.

	Correlation coefficient (*r*)	*P* Value	Regression equation
Trps1-E-cadherin	0.981	0.000	*t* = 0.596+0.42e
Trps1-CK19	0.913	0.002	*t* = 0.326+0.42c
Trps1-Vimentin	–0.711	0.009	*t* = 2.898–1.26v
Trps1-–α-SMA	–0.847	0.005	*t* = 1.505–1.13s

### Effects of Trps1 expression levels on EMT

**Fig 5:**
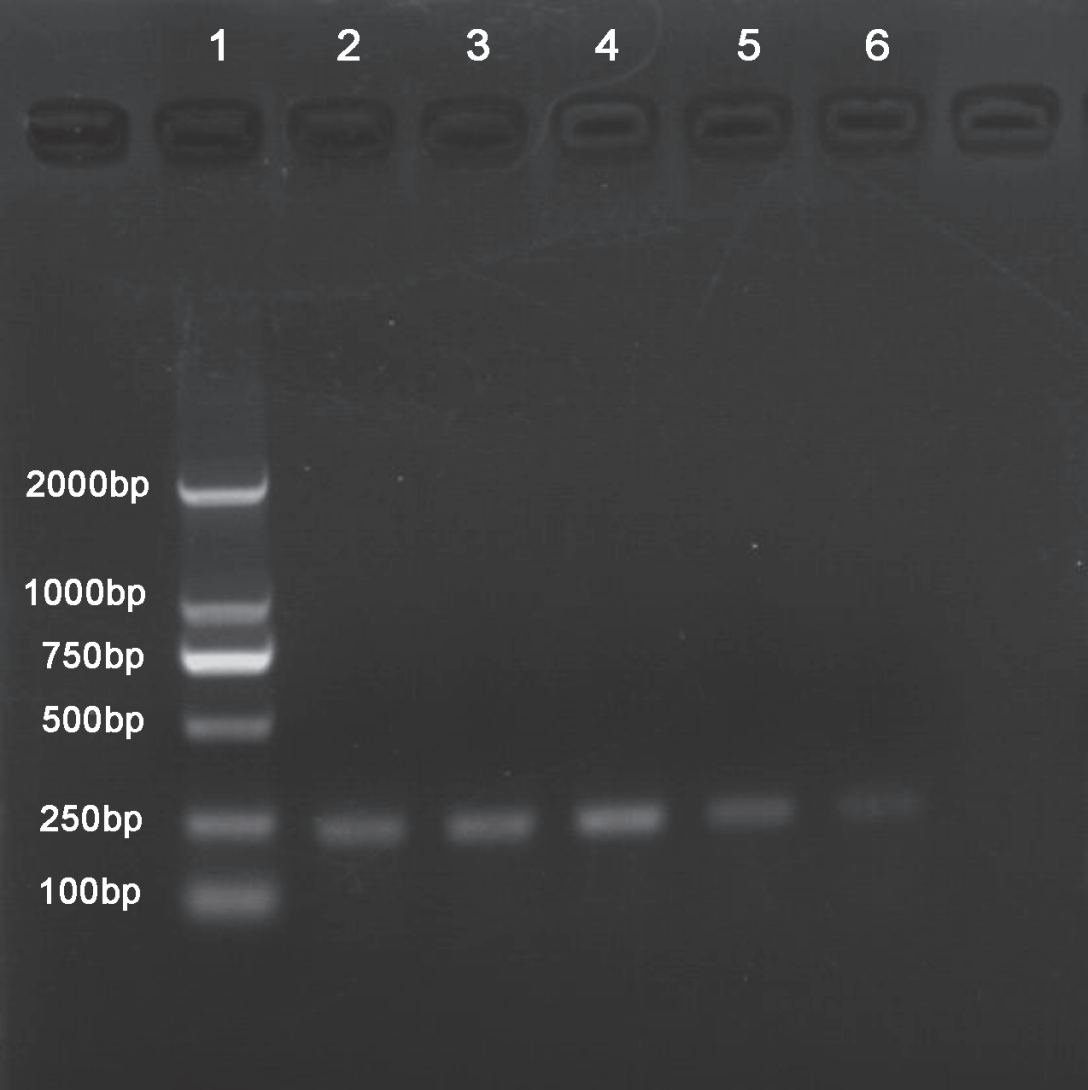
HIBECs were infected with a Trps1 adenovirus or transfected with a Trps1-specific siRNA. Lane 1: Marker2000; 2: untransfected HIBECs; 3: HIBECs infected with an empty control virus; 4: HIBECs infected with a Trps1 adenovirus; 5: HIBECs transfected with control siRNA; 6: HIBECs transfected with Trps1-specific siRNA.

**Fig 6:**
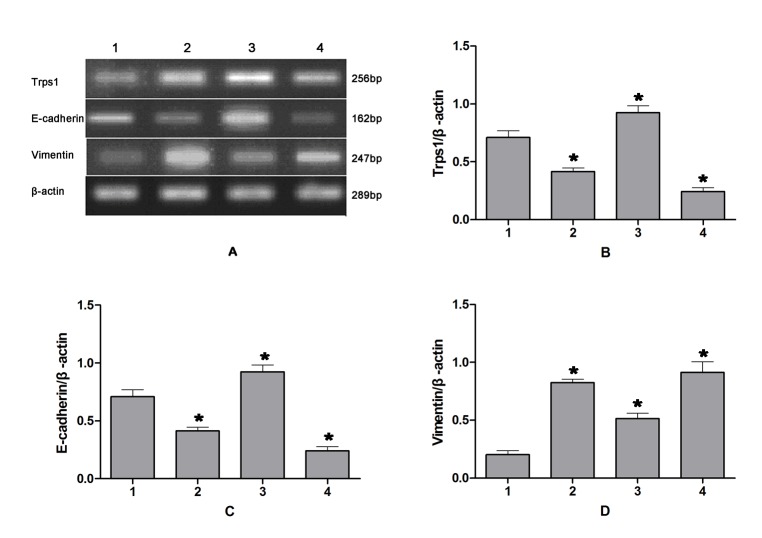
RT-PCR analysis of Trps1 expression changes during CPRI-mediated EMT in HIBECs. A: RT-PCR results; B–D: semi-quantitative Trps1, E-cadherin, and Vimentin mRNA results, respectively. Lane 1: HIBECs; 2: HIBECs subjected to CPRI; 3: HIBECs infected with a Trps1 adenovirus and subjected to CPRI; 4: HIBECs transfected with a Trps1-specific siRNA and subjected to CPRI.

**Fig 7:**
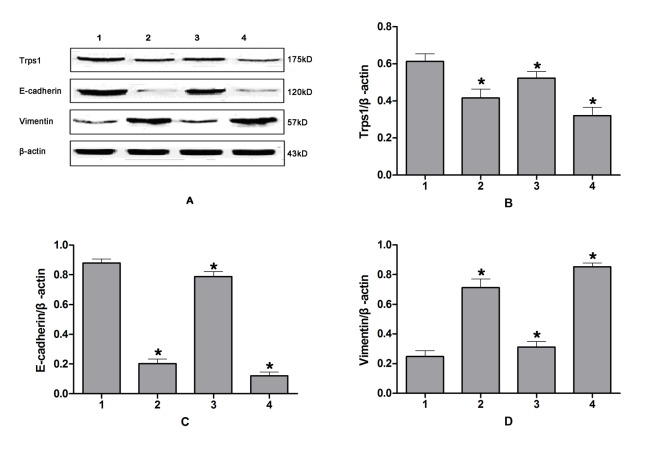
Western blotting analysis of Trps1 expression changes during CPRI-mediated EMT in HIBECs. A: Western blotting results; B–D: semi-quantitative Trps1, E-cadherin, and Vimentin protein results, respectively. Lane 1: HIBECs; 2: HIBECs subjected to CPRI; 3: HIBECs infected with a Trps1 adenoviurs and subjected to CPRI; 4: HIBECs transfected with a Trps1-specific siRNA, and subjected to CPRI.

Cells infected with the Trps1 adenovirus showed increased Trps1 mRNA levels compared with those in uninfected cells or cells infected with the empty control virus. When the infected HIBECs were subjected to CPRI, we observed alterations in the expression levels of genes associated with EMT. There was a significant increase in E-cadherin mRNA and protein levels (*p* = 0.002 and *p* < 0.001, respectively), and a significant decrease in Vimentin mRNA and protein levels (*p* < 0.001, *p* = 0.017), with CPRI-mediated EMT inhibited.Trps1 mRNA levels were decreased in cells transfected with the Trps1-specific siRNA compared with those in untransfected cells or those transfected with the control siRNA (Fig 5). In contrast, HIBECs transfected with the Trps1-specific siRNA and subjected to CPRI showed significantly decreased mRNA and protein expression levels for E-cadherin (*p* = 0.032 and 0.044, respectively). Vimentin mRNA and protein levels were significantly increased compared with those in untreated control cells (*p* = 0.047 and 0.031, respectively), with CPRI-mediated EMT enhanced (Figs 6 and 7).

## Discussion

EMT widely occurs in physiological and pathological processes such as embryonic development, organ formation, wound healing, organ fibrosis, and tumor metastasis. Many studies have confirmed that Trps1, a GATA family transcriptional factor, is involved in the regulation of EMT, and plays important roles in embryonic development and tumor metastasis. Kouros-mehr *et al*. [19] found that Trps1 was one of 22 transcription factors with high expression levels in the terminal end buds of the pubertal mammary ductal tree and the mature mammary ducts. Their results indicate that Trps1 plays important roles in the differentiation of luminal cells in the mammary gland. Malik *et al*. found that Trps1 mediated the differentiation of mesenchymal cells into distal respiratory epithelial cells, affecting the formation of the terminal respiratory tract during embryonic lung development. It has been shown that Trps1 knockout mice die because of neonatal respiratory failure. Gai *et al*. found that Trps1 mediated the differentiation of cap mesenchyme cells into renal tubular epithelial cells during renal development in mouse embryos. This stimulates ureteric bud branching, subsequently affecting the formation of renal tubules and glomeruli.

Recent studies investigating the relationship between Trps1 and tumor malignancy have revealed reduced cellular expression levels of Trps1 at the primary sites of endometrial carcinoma and breast, prostate, pancreatic, and colon cancers [20–24]. EMT was induced and cancer cells acquired the ability to migrate, thereby invading and disseminating to distant sites. However, the functions of Trps1 in wound healing and organ fibrosis have only been confirmed in the kidneys of experimental animals. Huang *et al*. [25] used a rat model of acute renal injury involving bilateral renal artery occlusion. They found that Trps1 expression was up-regulated during renal injury repair and negatively correlated with renal injury markers, indicating that it played roles in renal repair and regeneration. Gai *et al*. [26] used a model of unilateral ureteral obstruction in mice to generate kidney fibrosis. They confirmed that Trps1^+/-^ mice experienced stronger EMT than wild-type Trps1^+/+^ mice, supporting the hypothesis that Trps1 has a role in inhibiting EMT.

Using samples from NABS patients that underwent secondary liver transplantation, we found that Trps1 expression was either significantly reduced (*P* < 0.01) or absent when compared with that in normal bile duct tissues. Correlation between Trps1 changes in BECs and liver grafts, and expression of epithelial and mesenchymal markers confirm the involvement of Trps1 in the regulation of biliary EMT and fibrosis after liver transplantation, and therefore might play critical antagonist roles.

CPRI independently affects biliary fibrosis in liver grafts. Prolonged CP of liver grafts aggravates biliary endothelial damage during liver transplantation, and is a major reason for post-surgery biliary stricture and subsequent transplant complications [27–29]. A possible mechanism by which this occurs is CPRI-mediated EMT in BECs. Our findings confirm that BECs adopt mesenchymal characteristics following CPRI, an indicator that CPRI induces EMT in HIBECs. Prolonged CP (12–48 h) led to a more distinct form of EMT in BECs.

Our results regarding Trps1 expression patterns implies a role for Trps1 as a critical antagonist of EMT, and also suggests an important role for Trps1 in inhibiting and reversing the pathogenesis of organ fibrosis. Our results are consistent with the negative regulatory effects of Trps1 seen in other epithelial tissues and cells, where Trps1 expression initially increases and then gradually decreases. We postulate that increased Trps1 expression in BECs subjected to CP for less than 12 h might represent a compensatory process that protects cells from EMT progression. However, as the period of CP was prolonged, these compensatory effects declined and Trps1 expression levels gradually decreased, allowing for EMT to progress rapidly.

To the best of our knowledge, we are the first to confirm that Trps1 is involved in CPRI-mediated EMT in BECs, highlighting the antagonistic role of Trps1. CPRI inhibits Trps1 expression in BECs, subsequently stimulating EMT. This serves as an important factor in the development of biliary fibrosis in liver grafts. We propose that Trps1 represents a potential new target for the treatment of NABS that accompanies liver transplantation, however further research in alternative animal models is required.
